# Sagittal changes in the dens significantly slowed after 12 years of age

**DOI:** 10.1016/j.bas.2025.104233

**Published:** 2025-03-11

**Authors:** Zhi-Jun Sha, Sheng-Yu Fu, Zhao-Rui Wang, Hai-Feng Hang, Ai-Bing Huang

**Affiliations:** a Department of Spine Surgery, The Affiliated Taizhou People's Hospital of Nanjing Medical University, Taizhou, 225300, Jiangsu, China; b Department of Orthopedic, The First People's Hospital of Xianyang, Xianyang, 712000, Shaanxi, China; c Jiangdu People’s Hospital Affiliated to Yangzhou University, Yangzhou, 225000, Jiangsu, China

**Keywords:** Inclination, Odontoid, Pediatric, Growth, Cervical spine

## Abstract

**Introduction:**

The odontoid process is an important component of the upper cervical, and the process of ossification for odontoid does not cease completely until skeletal maturity.

**Research question:**

The aim of the study was to obtain the inclination parameters of the dens in many healthy children and to analyze the trends and variations in the inclination of the dens.

**Material and methods:**

All the CT data obtained from our hospital was reviewed for next measurement. Posterior edge of odontoid angle (PEOA), anterior edge of odontoid angle (AEOA), odontoid retroflection angle (ORA), posterior dens angulation angle (PDAA), screw insertion angle (SIA), and pB-C2 line were measured and analyzed.

**Results:**

A total of 219 patients were divided into 6 groups based on age at an interval of 3 years. The mean values of PEOA and PDAA dropped dramatically with age up to the ten to twelve-year group and then decreased slightly until 18 years old. Moreover, the AEOA and ORA declined gradually from birth to adulthood. These parameters were statistical significance within different age groups. However, the SIA was largely unchanged from birth to 18 years old and appeared to be independent of age. In contrast, the PB-C2 line has a distinguish distribution, with an increase up to the nine to twelve-year age group and then gradually decreased until 18 years old.

**Discussion and conclusion:**

The inclination of dens was constantly changing during pediatric growth, but the trends were different. These developmental changes slow down significantly after the age of 12 years.

## Highlights

• The dens is essential in the upper cervical region and is connected to the C2 vertebra via dentocentral synchondrosis.
• We undertook a more extensive investigation of the inclination of the dens to analyze its trends and variations during growth.
• The inclination of the dens constantly changed during pediatric growth, while these changes slow significantly after age 12.

## 1 Introduction

The dens is an important component of the upper cervical region, with the anterior atlas arch located anteriorly, the spinal cord and cranial fossa located posteriorly and superiorly, and the C2 vertebral body connected to it inferiorly by a dentocentral synchondrosis (Shane Tubbs et al., 2020; Akobo et al., 2015; Menezes, 2008). Numerous studies have shown that the primary and secondary ossification centers of the odontoid accompany the growth of the C2 vertebra during skeletal development (Menezes, 2008; Baaghtmyr, 2022; Piatt, 2011). These findings indicated that there are changes in the development of the dens with age.

Understanding its developmental changes can help surgeons better manage related disorders. Current literature focuses mainly on delineating the dimensions of the C2 morphology, and mostly on linear measurements in the transverse and coronal planes, while there is a paucity of data on developmental changes in the inclination of the dens (Piatt, 2011; Vachhrajani et al., 2014; Khaleel et al., 2014; Magat and Ozcan, 2019; Dou et al., 2021; Bapuraj et al., 2019; Stulik et al., 2021; Fu et al., 2022; Yin et al., 2024). In addition, the available data provide limited insight into whether retroversion of the dens is a risk factor for certain disorders (Chauhan et al., 2020; Koller et al., 2009; Ladner et al., 2015; Nowell and Nelson, 2019). Recently, in an anatomical study, Stulik et al. demonstrated that the posterior dens angulation angle (PDAA) transforms from anterior to posterior angulation during growth (Stulik et al., 2021). However, sagittal changes in the inclination of the dens in the pediatric population have not been systematically evaluated.

Therefore, we have undertaken a more extensive investigation of the inclination of the dens in a large number of children. We aimed to analyze its trends and variations during growth, thereby helping surgeons better understand changes in odontoid development in children to aid in surgical planning.

## 2 Methods

Patients aged 0–18 years who underwent CT examination of the craniocervical junction between 2016 and 2021 were retrospectively evaluated for inclusion in this cross-sectional study. The slice thickness for the image was 1 mm. Patients were excluded from the study if they had upper cervical trauma, tumors, prior spine surgery, cervical congenital malformation, or dysplasia. Moreover, patients whose CT images could not be reconstructed, or parameters could not be measured due to insufficient image resolution after reconstruction were also excluded. The project was approved by the Ethics Committee of Taizhou People 's Hospital (project number: KY2022-196-01), all data are not available to unauthorized individuals and raw data are not compiled.

To evaluate the odontoid process more accurately, the bony CT scan data (DICOM format) of patients were exported from the hospital picture archiving and communications system and then imported into Mimics software, version 17.0 (Materialise, Leuven, Belgium), for resegmentation. The coronal plane and transverse plane were rotated using the “online reslice” function to obtain the standard midsagittal plane.

**Fig. 1:**
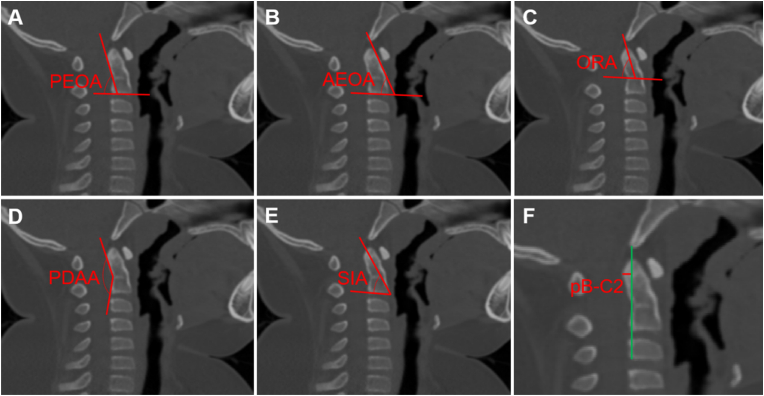
A, Posterior edge of the odontoid angle (PEOA): the angle between the posterior edge of the odontoid and the inferior endplate of C2; **B**, Anterior edge of the odontoid angle (AEOA): the angle between the anterior edge of the odontoid and the inferior endplate of C2; **C**, Odontoid retroflection angle (ORA); **D**, Posterior dens angulation angle (PDAA): the angle between the posterior edge of the odontoid and the C2 vertebrae; **E**, Screw insertion angle (SIA): the angle between the line from the superior posterior aspect of the odontoid to the inferior anterior aspect of C2 and the inferior endplate of C2; **F**, Perpendicular basion-C2 line (pB-C2): a line drawn through the posterior aspect of the odontoid perpendicular to a second line from the basion to the inferior posterior aspect of the C2 vertebra.

The following parameters were measured on the midsagittal plane: the posterior edge of the odontoid angle (PEOA), the angle between the posterior edge of the odontoid and the inferior endplate of C2; the anterior edge of the odontoid angle (AEOA), the angle between the anterior edge of the odontoid and the inferior endplate of C2; the odontoid retroflection angle (ORA), the angle between the line from the tip of the odontoid to the midpoint of the dentocentral synchondrosis and the line through the dentocentral synchondrosis; the posterior dens angulation angle (PDAA), the angle between the posterior edge of the odontoid and the C2 vertebrae; the screw insertion angle (SIA), the angle between the line from the superior posterior aspect of the odontoid to the inferior anterior aspect of C2 and the inferior endplate of C2; and the pB-C2 (perpendicular basion-C2 line), a line drawn through the posterior aspect of the odontoid perpendicular to a second line from the basion to the inferior posterior aspect of the C2 vertebra (Fig. 1).

The values measured were then used to calculate the means and standard deviations for each of the age groups and sexes. One-way ANOVA was performed for the age groups, and an independent samples *t*-test was performed for the sex groups. A P value < 0.05 indicated statistical significance. All the statistical analyses were performed using SPSS software (version 20.0, IBM, Armonk, NY, USA).

The intra/interobserver reliability was assessed by the intraclass correlation coefficient (ICC). For intraobserver repeatability, all the measurements were repeated by the primary observer for thirty randomly selected patients. For interobserver repeatability, all the measurements were repeated by an independent secondary observer for thirty randomly selected patients (a coefficient >0.8 was considered substantial, 0.65–0.8 was considered moderate, and <0.65 was considered poor).

## 3 Results

A total of 311 consecutive patients were identified for potential inclusion. Patients were excluded from our cohort for the following reasons: axial fracture, 4 patients; incomplete demographics, 5 patients; the inferior edge of the foramen magnum could not be displayed after rotation of the CT images in the software, 46 patients; and scan thickness greater than 1 mm, 37 patients.

A total of 219 patients who met the inclusion criteria were included. The average age of the children was 9.66 ± 5.04 years. Joseph H. Piatt Jr. et al. found that the neurocentral synchondroses and the dentocentral synchondrosis were generally no longer visible after 6 years, and the posterior midline synchondroses remained unfused until approximately the age of 3 years (Piatt and Grissom, 2011). Thus, for providing a relatively balanced distribution of the sample across different developmental stages, these patients were divided into 6 age groups by 3 years: group 1 (G1, 0–3 years old), group 2 (G2, 4–6 years old), group 3 (G3, 7–9 years old), group 4 (G4, 10–12 years old), group 5 (G5, 13–15 years old) and group 6 (G6, 16–18 years old) (Table 1).

**Table 1:** Demographics of patients according to age group and sex.

	Male	Female	Total
G1	14	15	29
G2	19	22	41
G3	22	19	41
G4	18	19	37
G5	18	18	36
G6	18	17	35
Total	109	110	219

G1:0–3 years old; G2: 4–6 years old; G3: 7–9 years old; G4: 10–12 years old; G5: 13–15 years old; G6: 16–18 years old.

The intra- and interobserver reliability ICCs of the parameters are listed in Table 2.

**Table 2:** Coefficients for interobserver and intraobserver measurement repeatability.

	Inter	Intra
PEOA	0.92	0.75
AEOA	0.64	0.79
ORA	0.81	0.84
PDAA	0.77	0.76
SIA	0.72	0.68
pB-C2	0.97	0.81

PEOA, posterior edge of odontoid angle; AEOA, anterior edge of odontoid angle; ORA, odontoid retroflection angle; PDAA, posterior dens angulation angle; SIA, screw insertion angle; pB-C2, perpendicular basion-C2 line.

The interobserver and intraobserver ICCs for measurement were acceptable (0.64–0.97 and 0.68 to 0.84, respectively).

The mean values of the parameters for the different sexes are shown in Table 3, and the results indicated that there were no statistically significant differences between the sexes.

**Table 3:** Comparison of the measured parameters between sexes (Mean ± SD).

	Male	Female	Total	P
PEOA (°)	72.75 ± 10.02	72.31 ± 10.33	72.53 ± 10.15	0.51
AEOA (°)	65.88 ± 5.32	65.84 ± 6.24	65.86 ± 5.78	0.27
ORA(°)	74.37 ± 5.03	73.61 ± 5.55	73.99 ± 5.30	0.42
PDAA(°)	164.29 ± 8.93	165.47 ± 9.02	164.88 ± 8.98	0.97
SIA(°)	59.15 ± 4.48	59.56 ± 4.77	59.35 ± 4.62	0.44
pB-C2(mm)	3.65 ± 1.62	4.27 ± 1.95	3.96 ± 1.82	0.12

PEOA, posterior edge of odontoid angle; AEOA, anterior edge of odontoid angle; ORA, odontoid retroflection angle; PDAA, posterior dens angulation angle; SIA, screw insertion angle; pB-C2, perpendicular basion-C2 line.

**Fig. 2:**
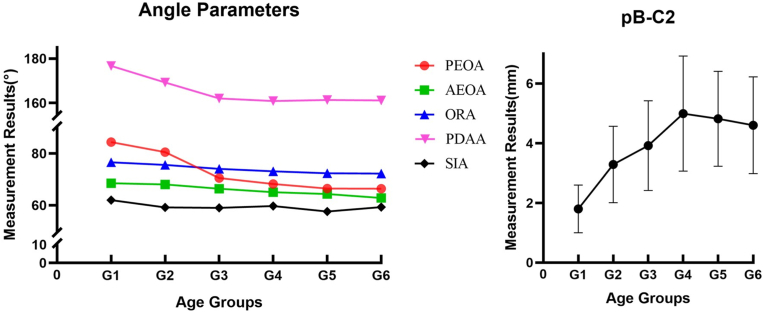
Left, PEOA: posterior edge of the odontoid angle; AEOA: anterior edge of the odontoid angle; ORA: odontoid retroflection angle; PDAA: posterior dens angulation angle; SIA: screw insertion angle. **Right,** pB-C2: perpendicular basion-C2 line.

The results of the measurements between age groups are shown in Table 4. Anatomical differences were evident among the 6 age groups, except for the SIA group. The PEOA decreased rapidly from 84.41 ± 10.08° in G1 to 70.54 ± 6.27° in G3 and then decreased slowly to 66.42 ± 6.65° in G6. A similar trend was observed for the PDAA; the mean PDAA decreased dramatically from 176.75 ± 9.91° in G1 to 162.07 ± 5.57° in G3 and then decreased slightly to 161.11 ± 7.43° in G6. Moreover, the mean value of AEOA decreased gradually from 68.55 ± 4.56° in G1 to 62.82 ± 6.25° in G6, which remained relatively constant. The ORA showed a similar distribution, with the mean value decreasing from 76.60 ± 7.55° in G1 to 72.26 ± 5.98° in G6. In contrast, the growth pattern of the pB-C2 line differed from that of all the other angular parameters, which gradually increased from 1.80 ± 0.80 mm in G1 to a peak of 4.99 ± 1.93 mm in G4 and then decreased to 4.60 ± 1.62 mm in G6. In addition, the SIA had a constant growth trend, although it decreased from 61.99 ± 3.98° in G1 to 58.99 ± 4.20° in G3. There was no statistical significance (Table 4, Fig. 2).

**Table 4:** Comparison of the parameters when divided into six age groups (Mean ± SD).

Age group	PEOA(°)	AEOA(°)	ORA(°)	PDAA(°)	SIA(°)	pB-C2 (mm)
G1	84.41 ± 10.08	68.55 ± 4.56	76.60 ± 7.55^a^	176.75 ± 9.91^a^	61.99 ± 3.98^a^	1.80 ± 0.80
G2	80.52 ± 8.35	68.06 ± 5.32	75.57 ± 4.29	169.24 ± 8.56	59.18 ± 5.15	3.29 ± 1.28
G3	70.54 ± 6.27	66.41 ± 5.26	74.09 ± 4.37	162.07 ± 5.57	58.99 ± 4.20	3.92 ± 1.50
G4	68.26 ± 7.04	65.04 ± 5.43	73.15 ± 4.70	160.87 ± 6.13	59.68 ± 4.59	4.99 ± 1.93
G5	66.46 ± 6.32	64.36 ± 5.93	72.36 ± 3.52	161.37 ± 4.42	57.56 ± 4.25	4.82 ± 1.59
G6	66.42 ± 6.65	62.82 ± 6.25	72.26 ± 5.98	161.11 ± 7.43	59.29 ± 4.62	4.60 ± 1.62
P	0.01	0.01	0.01	0.01	0.79	0.01

PEOA, posterior edge of odontoid angle; AEOA, anterior edge of odontoid angle; ORA, odontoid retroflection angle; PDAA, posterior dens angulation angle; SIA, screw insertion angle; pB-C2, perpendicular basion-C2 line.

a Some cases could not be measured.

## 4 Discussion

The morphology of the craniocervical junction has received much attention (Menezes, 2008; Vachhrajani et al., 2014; Dou et al., 2021). The dens, as the rotation axis of the atlantoaxial joint, is developmentally the most intricate of the cervical vertebrae and has always been the focus of research (Akobo et al., 2015; Ishak et al., 2020; O'Brien et al., 2015). Therefore, it is important for surgeons to understand the normal, variant and anomalous features of the odontoid to distinguish them from traumatic injuries for proper diagnosis. In the present study, engineering software was used to adjust the axial CT images, and standard midsagittal images were obtained to evaluate the inclination of the dens more accurately. The inclination of the dens constantly changed during pediatric growth, but the trends differed.

The morphology of the dens plays an important role in the stability of the atlantoaxial joint. Some scholars have studied the relationship between the odontoid process and atlantoaxial joint abnormalities (Bapuraj et al., 2019). Chauhan et al. noted that the degree of atlantoaxial dislocation is approximately 20° greater in children with atlantocervical inclination than in normal children (Chauhan et al., 2020). These findings indicated that retroversion of the anterior edge of the odontoid process was correlated with an increased incidence of atlantoaxial joint dislocation. Li et al. also suggested that the retroverted odontoid process was more likely to induce posterior atlantoaxial subluxation during cervical hyperextension (Li et al., 2022). To determine the inclination of the dens, a study using MRI of the C2 vertebra in pediatric Chiari I malformation patients revealed that the ORA decreased from 84.7 ± 10.7° in the 0- to 6-year-old group to 76.6 ± 11.7° in the 13- to 17-year-old group. This means that the odontoid process becomes more posteriorly inclined with increasing age in pediatric Chiari I malformation patients (Ladner et al., 2015). In another study, Tubbs et al. demonstrated that the ORA ranged from −89° to +101° in 50 normal children (Tubbs Pac et al., 2003). Recently, Jayapalli et al. attempted to establish parameters for normal pediatric spine growth during childhood in 1458 eligible patients. Their results showed that the dens angulation (SIA) gradually decreased from 81.27° ± 4.21° at age 1–71.85° ± 8.69° at age 18 in males and from 81.81° ± 2.72°–69.28° ± 3.90° in females (Bapuraj et al., 2019). These findings confirmed that the inclination of the odontoid process could change during pediatric growth. The limitations of the aforementioned study included a small sample size and the use of a single measurement metric. The developmental changes and variations in the inclination of the dens are unknown. In our study, we systematically analyzed the inclination of the dens and found that there were changes in morphology over the course of development. The PEOA decreased rapidly from 84.41 ± 10.08° in G1 to 70.54 ± 6.27° in G3 and then decreased slowly to 66.42 ± 6.65° in G6. Moreover, although the AEOA and ORA decreased gradually in G1 to G6, they both remained relatively constant during childhood with a similar distribution. Interestingly, the SIA did not change with advancing age in our study. It has a similar value to that reported in adults in the literature. This finding is contrary to that of a previous study that suggested that SIA changes with advancing age (Tubbs Pac et al., 2003).

The PDAA angle was first introduced by Štulík et al. The authors believed that understanding the PDAA holds potential for restoring the atlantoaxial complex to its correct position. In their anatomic study, the mean PDAA was 162.7° in males and 160.26° in females (Štulík et al., 2018). Shigeyuki et al. took a similar measurement and revealed that the inclination of the posterior edge of the odontoid process was −21.4° ± 23.3°. They emphasized that angled odontoid fractures with an angle of approximately 30° could remodel towards a normal morphological state at the time of bony union in patients aged less than 3 years (Tokunaga et al., 2011). Subsequently, the authors investigated the degree of the PDAA in healthy children and reported that the PDAA decreased from 200.65 ± 1.25 at 1 year old to 164.78 ± 5.65 at 16–18 years old (Stulik et al., 2021). They indicated that the dens no longer had anterior angulation but instead had posterior angulation, and change occurred between 4 and 6 years of age. In the present study, we found that the mean PDAA decreased dramatically from 176.75 ± 9.91° in G1 to 162.07 ± 5.57° in G3 and then decreased slightly to 161.11 ± 7.43° in G6. This means that the dens showed a straight line or even inclined forwards in G1; then, it underwent retroversion in G3 and remained steady until adulthood. Changes in development mainly occur before the age of 7.

The pB-C2 line was described by Grabb et al. for the quantitative description of compression of the odontoid process on the spinal canal, which represents the amount of ventral canal encroachment (Paul et al., 1999). Tubbs et al. measured the pB-C2 lines of 50 healthy children and reported that the range for the pB–C2 line ranged from −3 to 4.8 mm, with a mean value of 2.9 mm (Tubbs Pac et al., 2003). Using bony CT scans for pB–C2 measurements, Jayapalli et al. reported that the pB-C2 line increased with age and remained stable after 4–5 years of age until adulthood, and the maximum value for men was 6.71 mm ± 1.06 mm at the age of 10–11 years (Bapuraj et al., 2019). In the present study, the pB-C2 measurement increased from 1.80 ± 0.80 mm in the G1 age group to a peak of 4.99 ± 1.93 mm in the G4 age group and then decreased to 4.60 ± 1.62 mm in the G6 age group. It is imperative to consider these developmental changes when assessing the significance of pB–C2 values in the context of treating patients with ventral compression.

The ossification and fusion of the dentocentral synchondrosis occurs in individuals aged 6–11 years, while the growth of the axial vertebra does not cease until adulthood (Akobo et al., 2015). In the present study, the results demonstrated that the inclination of the dens significantly changed and varied before 12 years of age (before G4), which indicated that the increase in the inclination of the dens was attributed to dentocentral synchondrosis. Furthermore, no sex-based disparity is observed in this alteration. It is different from the dimensions of development changes in children, in which the dimension differences in growth acceleration are probably consistent with the periods of growth spurt (Koller et al., 2009; Segatto et al., 2014).

Shohat et al. reported 12 children with atrophic dysplasia and identified odontoid hypoplasia with subsequent subluxation of C1 and C2 as a common complication (Shohat et al., 1989). In addition, congenital deformity of the upper cervical spine is often associated with brain disease, so early identification of upper cervical spine developmental problems can help treat patients as early as possible to avoid pediatric orthopedic and neurological diseases (Smith, 1993). In light of this, greater familiarity with the normal developmental patterns of the dens will help ensure proper diagnosis, guide appropriate management for these patients and enable better preoperative planning. For example, with C1 lateral mass screw-C2 pedicle screw (C1LM-C2PS) fixation being considered the most stable fixation technique (Chun et al., 2018), the screw length and screw insertion angle should be more noteworthy for surgeons. We believe that there is an inseparable relationship between these parameters and the inclination of dens. Therefore, mastering the developmental trend of increase in the inclination of dens can help surgeons better grasp the relevant parameters of upper cervical spine fixation at all ages.

One of the strengths of this study was that engineering software was used to adjust the cervical CT to obtain standard midsagittal images for measurement, which can better help surgeons understand the developmental trends of the odontoid process in children. However, our study has limitations. First, this was a cross-sectional study, and the best choice would be a time-related study for pediatric growth. Second, the results of studies using only CT scans to conduct measurements may differ from those of other studies. In addition, the subjects in this study were all Asian, the results of which may not be applicable to Caucasian or other races.

## 5 Conclusion

The inclination of the dens constantly changed during pediatric growth, but the trends differed. These developmental changes slow significantly after age 12. For surgeons, this information is valuable for identifying the inclination of the dens in children and thus can better aid in preoperative planning in pediatric spine surgeries.

## Approvals

The Ethics Committee of Taizhou People’s Hospital Affiliated to Nanjing Medical University approved this retrospective study (IRB ID: KY202219601).

## Funding disclosure(s) statement

The author(s) received no financial support for the research, authorship, and/or publication of this article.

## Declaration of competing interest

The authors declare that they have no known competing financial interests or personal relationships that could have appeared to influence the work reported in this paper.
